# Farmers’ self-reported perceptions and behavioural impacts of a welfare scheme for suckler beef cattle in Ireland

**DOI:** 10.1186/2046-0481-66-1

**Published:** 2013-01-23

**Authors:** Andrea M Dwane, Simon J More, Martin Blake, Kenneth McKenzie, Alison J Hanlon

**Affiliations:** 1 UCD School of Veterinary Medicine, University College Dublin, Belfield, Dublin 4, Ireland; 2 UCD Centre for Veterinary Epidemiology and Risk Analysis, UCD School of Veterinary Medicine, University College Dublin, Belfield, Dublin 4, Ireland; 3 Department of Agriculture, Food and the Marine, Agriculture House, Kildare Street, Dublin 2, Ireland; 4 UCD School of Public Health, Physiotherapy and Population Science, University College Dublin, Belfield, Dublin 4, Ireland

**Keywords:** Animal welfare, Attitude, Beef cattle, Farmer behaviour, Focus groups, Ireland, Policy impact, Scheme

## Abstract

**Background:**

To date, there have been a limited number of studies on the impact of government-incentivised farm animal welfare programmes or ‘schemes’, and on farmers’ attitudes regarding such schemes. In this study, focus groups were used to gain insight into Irish farmers’ perceptions of such a scheme for suckler cattle and its behavioural impacts on farmers.

**Results:**

The findings were categorised into 46 codes and ultimately yielded two Global themes: 1) Beliefs and Evidence and 2) Logic and Logistics. The former theme covered farmers’ attitudes and observations regarding the Scheme. The latter dealt with factors such as workload and costs. The Global themes allowed for comprehensive reporting of the strongest messages from focus groups. There was consensus that Scheme measures for the minimum calving age and for weaning had a positive impact on welfare. Two aspects criticized by participants were firstly disbudding, due to the logistics for anaesthetic application, and secondly the administrative workload associated with data capture and utilisation. The majority anticipated that data being collected via the Scheme would help to inform farm management decisions in future.

**Conclusions:**

Farm animal welfare schemes, which incentivise participants to implement certain practices, aspire to long-term behavioural change after scheme conclusion. Our research showed that this Scheme increased farmer awareness of the benefits of certain practices. It also demonstrated the importance of stakeholder participation in the design stages of welfare initiatives to ensure scheme measures are practical and relevant, to address any perceived controversial measures, and to plan for training and adding value to schemes.

## Background

In 2008, Ireland’s Department of Agriculture, Fisheries and Food (DAFF; currently the Department of Agriculture, Food and the Marine (DAFM)) initiated a government-incentivised farm animal welfare programme entitled the “Animal Welfare, Recording and Breeding Scheme for Suckler Herds”, also known as the “Suckler Scheme". The Scheme’s aims were to enhance welfare standards and the genetic quality of the national beef herd and covered a range of on-farm inputs or ‘measures’ (see Table 1). This was a voluntary scheme based on financial incentives and it was designed to run for five years [1]. The scheme was funded entirely by the National Exchequer and required a major financial investment by the State. Scheme payments were originally set at €80 per eligible suckler cow but, due to budget cuts, this was reduced after one year to €40. Initial uptake was widespread with approximately 50,000 farmers (76% of registered suckler herds) enrolling. More recent figures showed approximately 55% of the national suckler herd still enrolled in 2011 [2].

**Table 1:** **Summary of the measures within the** ‘**animal welfare**, **recording and breeding scheme for suckler Herds**’ [3]

**Scheme measure**	**Specific requirements per measure**	
Calving details	Calf to be registered within 27 days of birth with details of sire and ‘ease of calving’ survey recorded.	
Disbudding of calves	To normally be carried out within 3 weeks of birth.	
	Illegal to disbud calf over 2 weeks old without using local anaesthesia.	
	Date of disbudding recorded.	
Castration of calves	Not compulsory.	
	If done, calf to be castrated at least 4 weeks prior to, or 2 weeks after weaning & date recorded.	
Minimum calving age	Average age of heifers calving for first time to be 24 months old.	
Appropriate weaning procedures	Minimum age for weaning set at 8 weeks of age.	
	3 different actions:	
	i.	Meal (concentrates) feeding from 4 weeks prior to weaning to 2 weeks post-weaning date
	ii.	Graduated weaning with calves weaned in at least two separate groups
	iii.	Sales Procedure with animals weaned a minimum of 2 weeks before sale or movement from herd
	Dates for actions recorded.	
Animal Events Recording	Farmers to submit all required data for each measure in the Scheme.	
Training and education	Farmers to attend a specified training course.	

Source: DAFM, (2009) [3].

The cost-effectiveness of a scheme can be assessed in monetary terms [2], and also in terms of its long-term influence on farmers’ behaviour and attitudes toward production systems that support good welfare. To date, there have been a limited number of studies on the impact of government-incentivised schemes to enhance farm animal welfare, and particularly on farmers’ attitudes to such animal welfare interventions [4]. This is in spite of the fact that it is often the policies and attitudes at farm level that may have a key effect on welfare. Hemsworth noted that a stockperson’s attitudes and behaviours can directly affect welfare [5]. In their study of stakeholder attitudes to farm animal welfare, Heleski et al. identified producer attitudes, economics and tradition as potential obstacles to enhancing welfare [6]. Fraser’s study of a range of animal welfare assurance programmes concluded that a programme’s effectiveness is dependent on several factors including the degree of support from producers and the ease of instituting and maintaining such programmes [7]. A review of the literature indicated that research on humans’ attitudes to animal welfare often targeted veterinary surgeons, animal science students, and consumers, rather than farmers [4,6]. Studies that did seek farmers’ opinions have predominantly focused on more intensive farming systems such as pig and dairy farming but not suckler beef systems [4,8]. The objectives of this study were to seek farmers’ opinions of the Scheme, to explore the underlying attitudes towards suckler herd welfare, and to elicit ideas that may inform the design of future schemes.

## Methods

### Convening the focus group

In this study, focus groups were used to gain an insight into Irish beef farmers’ perceptions of the ‘Suckler Scheme' and its behavioural impacts. The focus group format was piloted and ethical approval for the study was obtained from University College Dublin’s (UCD) Human Research Ethics Committee on 29^th^ June 2009 (Ref: LS-E-09-101-Dwane-Hanlon).

Four focus groups were conducted in November 2009. The groups comprised between 7 and 9 participants in four regions of the Republic of Ireland:

· Galway /West (W): 8 male farmers

· Donegal /North (N): 6 male and 1 female farmers

· Cork /South (S): 8 male farmers

· Kilkenny /East (E): 7 male and 2 female farmers

In total, 32 individuals participated in the study. Participants were required to be adult suckler beef farmers and were recruited irrespective of their age, gender or Scheme participation.

Participants were sourced through local private veterinary practitioners (PVPs). PVPs were briefed on the project and requested to approach suckler farmer clients and ask permission to pass on their contact details to the researcher. The researcher made contact with the farmers using a standardised invitation and recruitment procedure (Appendix 1). At the end of the focus groups, participants were given a €25 voucher to offset the cost of travel.

Focus groups lasted approximately 60 minutes and were facilitated by a recorder (scribe) and a discussion facilitator. The format of the discussion was explained to participants and they were requested to sign a consent form (further details available on request). All discussions were recorded on a digital voice recorder. The scribe was present in order to take field notes of the discussions, allowing for contextual elements of the focus groups to be captured (e.g. when there was strong consensus or feelings expressed by the group).

### Interview guide

The topic guide for the discussion (Appendix 2) consisted of three sections:

· Firstly, participants were requested to write down the three main risks to welfare for suckler cattle in Ireland. The group’s three most frequently recorded welfare risks were explored through facilitated group discussion.

· The second section explored participants’ attitudes towards the ‘Suckler Scheme'. Participants were given a list of the Scheme measures and asked to devise a hypothetical new scheme saying which of the existing measures they would opt to continue, refine or remove. The reasons for their decisions were explored. Participants were also invited to suggest additional measures that could be included in a new scheme.

· The final section focused on training, sources of information and learning resources.

### Qualitative data analysis

All audio recordings of the focus groups were transcribed *verbatim*. The full transcripts were then ‘coded’ separately by two members of the research team. Coding involved examining the transcripts and assigning a title (code) for each segment of the transcript to encapsulate the topic under discussion. Encoding the information organises the data so as to identify and develop themes from the codes [9]. In total, 46 codes were identified and agreed upon by the researchers (Table 2). The code list was used to structure the transcripts of all focus group discussions.

**Table 2:** List of all 46 codes used during thematic analysis of focus group transcripts

1	Age at first calving
2	Age of the cow
3	Breeds/ breed selection
4	Sire bulls and bull ratings
5	Bull beef
6	Buying in calves / weanlings
7	Buying in replacements (heifers)
8	Calving
9	Castration
10	Characteristics of suckler farmers
11	Cleanliness
12	Colostrum
13	Costs for resources (eg veterinary)
14	Culling
15	Disbudding/ Scheme re disbudding
16	Disease of herd and biosecurity
17	Dosing of calves (for hoose, etc.)
18	Drying off the cow
19	European market vs. home market
20	Exercise for cows
21	Expectations for the future
22	Farmers asking for information from each other
23	Mineral supplements for cows
24	Feeding the cow / cow body condition pre- and post-calving
25	Financial payments and outgoings
26	Handling suckler cattle
27	Housing
28	Improving the Scheme
29	The Mart
30	Meal-feeding
31	Medicines
32	Numbers of animals/ stocking density
33	Paperwork
34	Polledness
35	Pricing of beef
36	Rural Environmental Protection Scheme (REPS) / Cross compliance between Schemes
37	Scheme payments
38	Seasonal effects
39	Strong agreement on a topic within the group
40	Traceability of animals
41	Training and education
42	Type of land
43	Vaccinations
44	Vets
45	Viruses in calves
46	Weaning procedures

Thematic analysis involves coding followed by the creation of Organising and Global themes [9]. ‘Organising themes’ encompass a number of codes in such a way as to express their common sentiment or idea. ‘Global themes’ are the overarching themes to encompass a number of the Organising themes so that data may be presented in a logical and systematic manner. Coding and thematic analysis were carried out using NVIVO 8, a software programme designed for qualitative data analysis.

**Figure 1:**
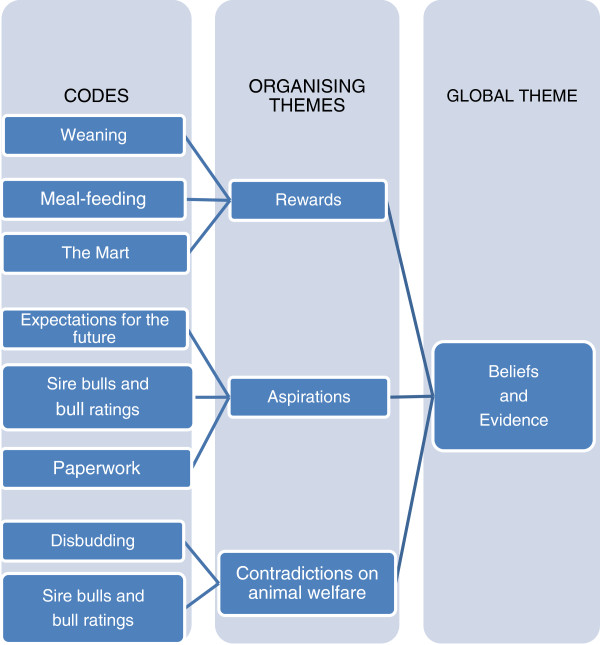
Examples of thematic analysis of focus group data for the Global theme “Beliefs and Evidence”.

**Figure 2:**
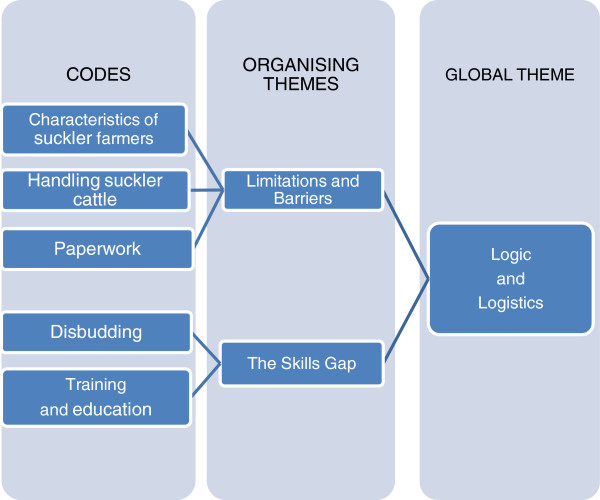
Examples of thematic analysis of focus group data for the Global Theme “Logic and Logistics”.

Following coding, the data was ordered first into ‘Organising themes’. As one example, in the case of three codes “Paperwork”, “Sire bulls and bull ratings” and “Expectations for the future”, focus group participants had expressed the belief that the information being gathered under the Scheme regarding bull sires and calving difficulties (dystocia) would be of value to farmers in the future. These codes therefore fed into the Organising theme of “Aspirations” (Figure 1). In total, five Organising themes were identified: “Aspirations”, “Rewards”, “Contradictions on animal welfare”, “Limitations and barriers” and “The skills gap”. Analysis of the Organising themes was undertaken to identify the Global themes and revealed that the former three Organising themes fed into a Global theme of “Beliefs and Evidence” (example in Figure 1) and the latter two pertained to a second Global theme of “Logic and Logistics” (example in Figure 2).

## Results

Thematic analysis resulted in 46 codes (Table 2) with five Organising themes which on analysis were determined to fit into the two following Global themes (see Figures 1 and 2):

1. **Beliefs and Evidence**

2. **Logic and Logistics**

“**Beliefs and Evidence**” pertains to farmers’ beliefs in relation to the Scheme and its impact on farmer behaviour. It contains three (Organising) sub-themes, ‘Rewards’, ‘Aspirations’ and ‘Contradictions on animal welfare’.

### i. Rewards

Rewards reflect aspects of the Scheme which farmers believed to have been rewarding, either financially or in terms of the well-being of their stock. Meal-feeding in advance of weaning (‘Appropriate weaning procedures’ measure) was the criterion that the majority of participants (focus groups N, E and W) described as being especially rewarding. Participants commented on how it had reduced the stress of weaning and resulted in stronger and more docile calves.

To quote one farmer (E):

"“They (the calves) certainly could do with a bit of meal before starting out (with weaning) and certainly the Scheme was good for that … (to) take some of the stress away from them.”"

Another aspect of this measure recognised (N, S & E) for having reduced the stress of weaning was the gradual separation of calves and cows.

Some participants however expressed dissatisfaction with aspects of the ‘Weaning’ measure, namely the stipulation that calves may not be moved for sale until two weeks post-weaning.

The reason stated by participants for this dissatisfaction was financial loss at sale:

"“If you go to any of the sales …the lads that are weaning are losing possibly up to €100 a calf by having them weaned on time, properly.” (W)"

### ii. Aspirations

This sub-theme pertains to measures that farmers believed would have long term benefits. While participants expressed frustration with the large volumes of Scheme paperwork, the majority (N, E & W) conveyed high expectations for the future (once all data on the Scheme forms has been analysed). However they also expressed dismay that such results were not becoming available more rapidly.

As one farmer (N) explained:

"“The recording… all (goes) to ICBF (Irish Cattle Breeding Federation)… so it should be beneficial… in the years to come.”"

Another (E) commented:

"“Now everyone is putting down in a box if they (the calvings) are easy or… hard….but there is no feedback …to say if there is… (genetic) strains… giving terrible trouble (with calving)….We are getting no information… It’s a blank canvas going looking for a bull.”"

In general, there was a positive feeling that the Scheme data would generate information of benefit to farmers in the future.

### iii. Contradictions on animal welfare

This sub-theme pertains to Scheme measures that farmers believed to be unrelated to, or adversely impacting on animal welfare. Scheme measures on ‘Minimum calving age’, ‘Weaning’, ‘Disbudding’, and ‘Castration’ were all considered directly relevant to welfare, but only the former two were reported by participants as having a positive impact on welfare (S, W & E).

Several farmers (S, W & E) believed that the ‘Disbudding’ measure had negatively impacted on animal welfare and questioned its rationale. Farmers explained that horn bud growth may not occur until 3–4 weeks of age. Also, farmers appeared reluctant to administer local anaesthetic and unconvinced of its benefits. Therefore, in order to comply with the Scheme and avoid using local anaesthetic, participants (S, E & W) indicated that farmers may try to disbud calves with no detectable horn bud.

As one farmer (S) explained:

"“You would find something and you would be wondering: Is that a horn now or is it not? You could be polling (disbudding) calves that you know you are guessing … just to satisfy the Scheme.”"

The majority suggested that the age limits should be extended:

"“What’s the difference… between 3… and 6 weeks? What has changed other than…that you can see the horn…? I would say it (disbudding) is less sore on a stronger one (calf) than on a little one.” (W)"

Another area of contradiction noted in terms of animal welfare was the promotion of large purebred bull sires at Scheme training days:

"“They were showing that (large bulls) at a lecture on animal welfare … But there was no mention of animal welfare at all in the cow calving… and …that could be the biggest (welfare) problem for most farmers… there is no emphasis at all on easy calving sires… I don’t know how …he (the bull) is easy-calving when he’s hardly able to come in the door.” (W)"

The second Global theme “**Logic and Logistics**” relates to factors that impacted on farmers’ ability to implement Scheme measures. It contains two sub-themes, ‘Limitations and barriers’ and ‘The skills gap’.

### i. Limitations and barriers

This sub-theme pertains to the daily routine on-farm and also to external influences which impacted on how farmers applied Scheme measures.

The 50% reduction in Scheme payments in 2009 (from €80 to €40 per cow) was discussed and for many participants (S, E & W), their discussions reflected a belief that Scheme participation was no longer an economically viable option:

"“€80 wasn’t too bad …it was nearly covering (costs) but €40…you couldn’t do it…you’d be losing money.” (W)"

The volume of Scheme paperwork was an issue for participants (W & E):

"“My son does it online; only for that, I wouldn’t do it because they (DAFM) are sending you back paperwork every day…” (E)"

The forms for ‘Animal Events Recording’ were perceived as being particularly detailed and difficult to fill in.

Another consideration was the manpower available on–farm to assist with handling animals.

As one farmer (W) explained in relation to ‘Disbudding’:

"“You can’t be disturbing cows every 2 to 3 days…there’s no help on a lot of farms now…Are you going to be bringing in help every week to do (disbud) 3 or 4 or 5 calves whereas when you can bring them in and do 20 calves (at the one time)?”"

### ii. The skills gap

This sub-theme pertains to the differences between the skills required to satisfy Scheme criteria, the farmers’ existing skillset, and the Scheme training provided. Some participants (S, W & E) indicated that farmers were not using local anaesthetic when disbudding calves and elected to disbud under two weeks of age (irrespective of whether the horn bud was palpable or not) in order to avoid the anaesthetic requirement.

Comments from participants suggested there is uncertainty regarding the correct technique for administering the anaesthetic, with the following comment illustrating the erroneous belief that an intravenous injection is required:

"“I would prefer just to go on and get the job done…I would be more uneasy trying to find a vein, messing around with a needle.” (S)"

None of the participants spoke of having personally used local anaesthetic when disbudding. Although local anaesthetic techniques for disbudding had formed part of the Scheme training, none of the participants mentioned this aspect of the training.

When asked about the mandatory Scheme training, two groups (N & W) responded that people attended only because it was required to receive payments. Some also stated that all they wanted from training was information on the Scheme paperwork. A number of farmers felt the training was too basic. Conversely, some older focus group participants (50 years +) mentioned that they had found it helpful in order to ‘brush up’ on techniques.

## Discussion

The aims of this study were to seek farmers’ opinions of the current Scheme, to explore their underlying attitudes, and to elicit ideas to inform future scheme design. Data collected via focus groups yielded two global themes. Within the theme “Beliefs and Evidence” were included farmers’ perceptions regarding the Scheme, which were generally positive in relation to Scheme measures but appeared conflicted or negative regarding disbudding, large sire bulls, and record-keeping administration. The theme “Logic and Logistics” covered day-to-day parameters such as financial considerations. In the first part of this section, key opinions and attitudes captured during the focus groups are discussed and, in the second part, the study methods are examined and their impact considered.

### Discussion of key focus group findings

Policy-makers and those involved in the design of welfare initiatives need to be alert to the possibility of a ‘mismatch’ occurring between initiatives and farmers’ attitudes. This was evident for the ‘Disbudding’ measure and also for the Scheme’s promotion of sire bulls.

Mismatches between welfare initiatives and farmers’ attitudes may lead to non-compliance, or worse may inadvertently result in a negative impact on welfare. It appeared that the ‘norm’ was not to use local anaesthetic when disbudding. Similar findings were also reported in a study of Canadian beef farmers [10]. Focus group participants expressed the opinion that the Scheme needed to be altered to allow farmers to disbud older calves without anaesthetic and appeared unaware that this Scheme measure conforms with national legislation pertaining to all calves. There was no interest expressed in learning local anaesthetic techniques. Farmers reported that in an attempt to comply with the Scheme and avoid using local anaesthetic, calves less than two weeks old were being disbudded even if there were no detectable horn buds. While the ‘Disbudding’ measure may have been beneficial in reducing the incidence of dehorning of adult cattle [2], it appears that the Scheme measure and resultant farmer behaviour may have had an unanticipated adverse impact on calf welfare. Early disbudding may be inaccurate with horn regrowth, meaning the calves may need to be subjected to the procedure a second time. It is widely accepted that beef calves can be over two weeks of age before any buds develop and our findings suggest that the age criteria for disbudding appeared illogical to farmers. Furthermore, there seem to be inconsistencies between research findings and the current guidance on disbudding. Research indicates that young calves may be equally or possibly more sensitive to pain than older calves [11], supporting the farmers’ opinion that older calves are better able to withstand the pain of disbudding than younger calves and raising doubts about the validity of age-related anaesthetic guidelines

Whilst other factors (e.g. manpower and training/ skills issues) may have affected disbudding behaviours, it is likely that the farmers’ attitudes and the perceived inconsistencies in the underlying rationale were major barriers to behavioural change. Findings in other studies suggest that when farmers experience a mismatch, they need a “trusted source to advise them… before they would consider any action” [12]. It is suggested that if the conflict relating to the ‘Disbudding’ measure had been identified in advance, possibly during Scheme piloting, then training could have been adapted to address the disbudding anaesthetic techniques more comprehensively. A previous study on farmer behaviour noted that “knowledge of what motivates or inhibits farmer behaviour… will aid policy-makers… to target specific issues and maximize the effect of the control measure(s)” [12].

Participants considered the promotion of large sire bulls at Scheme training sessions as paradoxical within the remit of promoting good welfare, because of the implications associated with dystocia. Apart from the ‘Minimum calving age’ measure (which the farmers strongly supported as being beneficial to welfare), the Scheme contained no measure to prevent dystocia. There are a number of possible reasons for this, most notably a lack of existing data on the incidence of dystocia in suckler herds (i.e. the problem is not quantifiable as yet). In order to address a welfare issue “the problem must first be quantifiable and any measures put in place (in order to combat the risk) must be measurable” [13]. The Scheme’s ‘Animal Events Recording’ did include an ‘ease of calving’ survey, which means data collection on sire bulls and dystocia has commenced. Farmers acknowledged the timescale required for generating information from this captured data. The Scheme review noted that breeding data for the dairy industry (dairy’s Economic Breeding Index (EBI)) took approximately six years to have a noticeable impact and it was anticipated that Scheme data on calving would not have a noticeable benefit “for a number of years” [2].

Financial considerations impact greatly on farm management. A study of European state-sponsored environmental initiatives in 2000 stated that farmers willingly adopted practices with a clear financial benefit but also cautioned that there was no evidence that this resulted in any fundamental longer term change in their underlying values or practices [14]. Farmers may have positive attitudes to the Scheme and/or welfare but if this does not equate with running an efficient and profitable farm business then it becomes most unlikely for the farmers to behave in accordance with their personal beliefs. While financial reasons are not always at the root of welfare issues, our findings indicate that such reasons did have a role in farmers’ decisions in relation to practices such as meal-feeding and the timing for moving weaned calves to sale. Whilst farmers recognised that changes in these practices may improve animal welfare, implementing change was considered counterintuitive if by doing so they would incur financial losses. According to DAFM, Scheme payments were originally calculated at €80 to address the potential costs associated with additional handling, meal-feeding and delayed movement to sales [Personal communication, 8 October 2008]. Our findings suggest that according to participants, the additional costs of Scheme participation were acceptable when payments were at €80 per cow but it was no longer perceived as a viable option for many when payments were halved. This would concur with the calculations which determined the appropriate initial Scheme payment of €80 per cow. It is likely that the reduced numbers of farmers taking part in the Scheme in more recent times reflects this financial concern [2].

According to a 2011 survey of Scheme participants, 63% of the 170 farmers surveyed said the payments did not cover the costs of participation [2]. 52% of those surveyed indicated that they had experienced increased profits at sales of calves [2], which contradicts the focus group finding that weaning in advance of sale was causing a loss to farmers. This contradiction may be due to the fact that market prices have been steadily improving since the focus groups were conducted in 2009 [2]. The survey results were more recent and would therefore better reflect the price increase. The survey findings indicated that 56% of farmers would continue weaning in advance of sales [2]. To summarise, these survey results indicate that approximately half of Scheme participants may be receiving better prices at weanling sales and would continue the Scheme ‘Weaning’ measure.

The financial viability of participating in welfare initiatives is an ongoing concern and one that was highlighted in the focus groups. It may be argued that farmers’ answers to questions regarding Scheme payments may be motivated by a desire to ensure financial payments remain in place and may not reflect the actual circumstances. The downturn in the Irish economy and resulting reduction in Scheme payments in 2009 had a noticeable impact on participation albeit less of an impact than may have been initially feared [2]. Main suggested that schemes could aim to maximise profitability as well as improve welfare by demonstrating and publicising farmers’ compliance with welfare guidelines and by maintaining and developing markets for farm produce [15]. Focus group participants alluded to the lack of information available when purchasing cattle in mart sales and there was a sense that if wider publicity were given for cattle reared under the Scheme, this may certainly influence buyers and ultimately more farmers to become involved in welfare initiatives such as the current Scheme. According to DAFM, marts have started in more recent times to display details of Scheme membership at point of sale [Personal communication, 9 December 2012].

### Discussion of the study methods

Focus groups were the chosen method for data collection as this approach is useful for exploring people's knowledge and experiences, and to examine not only what people think but the underlying reasons why they think that way [16]. Focus groups provide qualitative data from sub-populations within a community. However, focus group composition and output may not necessarily be representative for the overall population. Determining the appropriate number of focus groups may be dependent on resources, but ideally they should continue until no further new ideas are being generated, that is until a ‘saturation point’ is reached. This point is commonly achieved with between four and six focus groups [17]. In this study, there was a high level of consensus and repetition of opinions and attitudes expressed in the four groups regarding the Scheme, which suggests that the saturation point was reached.

Careful consideration was given to the recruitment of focus group participants. A well-designed focus group usually consists of between 6 and 12 participants [18]. Geographical distribution with the convening of an adequate number of groups to allow “the group to consist of representative members of the larger population” [19] was a consideration the researchers sought to satisfy. Recruitment via local PVPs worked well and with the exception of three farmers who had personal commitments or reasons for not attending, uptake was high when invited to participate in the focus groups. It is acknowledged that the ‘filter’ applied by the PVPs meant that participants were not randomly selected. There was an inherent bias in convening a focus group by this method as recruiters (PVPs) were likely to invite those most likely to agree to participate. Also it is accepted that certain individuals may be more inclined to agree to discuss animal welfare. Conversely, it is worth noting that, if it is assumed that the focus group participants are likely to be those more interested in animal welfare, this may mean that the focus group findings reflect the behaviours of individuals most motivated to improve their animals’ welfare and comply with a welfare scheme.

Due to ethical considerations of the group dynamic, focus group participants were not asked to divulge personal information such as age nor participation/ membership status in relation to the Scheme. All participants were familiar with Scheme measures and willing to discuss them. However, our study was unable to capture differences of opinion between Scheme participants and non-participants.

The researchers were aware that the 2009 reductions in Scheme payments were likely to affect the November 2009 focus group results. In order to facilitate discussion of the Scheme measures outside of a solely financial context, participants were asked hypothetical questions about creating a new scheme (see Appendix 2). Participants engaged with these questions although discussions of financial implications did inevitably occur. However, this approach permitted the exploration of welfare measures beyond the limit of financial considerations and highlighted the importance of careful question selection and discussion facilitation.

## Conclusions

Farm animal welfare schemes which incentivise participants to implement certain practices aspire to long-term behavioural change after a scheme’s conclusion. There are a number of attitudinal factors which may influence the successful implementation of a government-incentivised welfare scheme. Whay observed that a successful intervention requires the implementers (i.e. the farmers) to be motivated to change their practices [20]. Findings from this study showed that the Scheme increased farmer awareness of the benefits of certain new practices and suggested that farmers will be incentivised to continue certain practices after it ends, namely the minimum calving age, meal-feeding in advance of weaning and gradual separation of cows from calves at weaning. Our findings relating to the Scheme’s long-term behavioural impacts are largely supported by findings from a recent review [2]. However our results highlighted the challenges faced when improvements in welfare are perceived to be financially-, or logistically-questionable, as well as the difficulties involved in providing appropriate education to large cohorts of farmers of differing ages and/or prior training. Our research also demonstrated the importance of stakeholder participation in the design stage of welfare initiatives to ensure that scheme measures are practical and relevant, to anticipate and address any perceived contradictory measures, to inform the focus of any related training, and to seek ways to add value to schemes, possibly in terms of public opinion and/ or market share.

## Appendix 1: Supplementary information re: focus group recruitment and invitation letter

**Recruitment procedure**:

· Step 1 (Acquiring contact details):

Researcher ↔ Local veterinary surgeon ↔ Beef farmer

· Step 2 (Verbal invitation):

Researcher → Beef farmer

· Step 3 (Written invitation):

Researcher → Beef farmer

**Invitation letter**:

***Re***: ***Focus Groups discussing Farmers***’ ***Perceptions of Farm Animal Welfare***

Dear ____________________________,

Following our introduction by (vet), I write to invite you to partake in the first ever Irish focus group discussions on farmers’ perceptions of the welfare of beef suckler cattle. You are invited as an Irish suckler farmer to participate in the discussions. This study is being led by the School of Agriculture, Food Science & Veterinary Medicine in University College Dublin (UCD).

This focus group meeting in your area will take place in the **Teagasc facility in** __**from 10**.**30am**- **12**.**00 pm** (**meeting will run from 11**–**12**) **on** (**day**) (**date**) **November 2009**. The help of Mr. ____, from Teagasc in ___, in arranging these meetings is gratefully acknowledged. Refreshments will be provided and a ‘One for All’ voucher for the value of €25 will be given to each participant as a token of thanks for his/her participation. The actual discussion will run for a maximum of 60 minutes. In attendance will be a maximum of 7–9 other suckler farmers and two UCD employees: the postgraduate student conducting the research (Andrea Dwane, qualified as vet from UCD in 1996, now a lecturer in UCD; tel: __; mobile: __; e-mail: andrea.dwane@ucd.ie ) and one other UCD staff member (the research project supervisor, Dr. Alison Hanlon; e-mail: __). **This focus group meeting will be totally confidential**, **and any individual information collected will be made anonymous and summarised to produce group data for this study only**.

We would really appreciate your cooperation with this project. Results from this research will be reviewed by the Department of Agriculture in order to inform future developments in national policy regarding farm animal welfare. Therefore, **the collective views of Irish suckler farmers are vital** in identifying the welfare issues that are of real concern to farmers and in reviewing the mechanisms employed to improve beef suckler cattle welfare.

I will contact you in the coming days to confirm your attendance at the focus group discussion.

Sincere thanks in advance for your co-operation on behalf of the research team.

Andrea Dwane

## Appendix 2: Interview guide for focus groups

**Question**/ **Discussion Guide** &**Draft** ‘**Script**’ **for Focus Groups**:

**Total Participant time required**: **50**–**60 minutes**

Total focus group time: 50–60 minutes

Equipment: Consent forms & info sheets, Index cards, Post-it notes, digital recorders x 2 , batteries as required, laminated Scheme information sheets (see Table 3), scribe/ facilitator, writing materials/ pens, facilitator’s notes, flip chart and markers.

**Table 3:** Draft of the Laminated Table supplied for Focus Group Participants to view

Measures from ‘Suckler Scheme’	
·	**Registering of animal details**
·	**Disbudding of calves**
·	**Castration of calves**
·	**Age at first calving**
·	**Weaning procedure**
·	**Recording of Animal Events**
·	**Training**/ **education**

Specific objectives

· To identify key welfare issues relating to beef suckler herds in Ireland

· To explore and clarify the attitudes/ thinking behind the key issues raised

· To discuss mechanisms to promote animal welfare-friendly farming practices using stop/ start/ continue questions

"Focus group findings will be analysed for recurring themes and for comparisons between the various focus groupings."

Below is a draft of a general guide for leading the focus groups. This guide may be modified as needed as each focus group will inform the subsequent groups.

Before the group begins, conduct the informed consent process, and provide all necessary information (fire exits, breaks, etc.).

I. **Introduction**, **signing consent forms** &**explanation**/ **summary of study aims** (**3 min**)

II. **Topic Generation and Exploration** (**15 min**)

· The initial question (participants to be allowed to use Post-It notes to list their answers):

· *Today we are here to talk about the welfare of beef suckler herds*. *What three key areas of concern come to mind when you think about the welfare of Irish beef suckler herds*? *Please write your answers on the Post*-*It note sheet provided to you*.

· The group will provide a list of initial topics.

· The focus group facilitator will then:

Take the 2–3 main topics brought up and prompt the participants for more information/ exploration of attitudes:

► *e*.*g*. ____________________ *was mentioned a lot*. *Tell me why this is an important concern*.

► Other exploration questions may include: *Why this issue arises*? *Why it is significant for the cattle* / *for the herd owner*? *What is it about this issue that prevents it being resolved*? *What other factors do you think may be involved here*? *What are the barriers to improving the animal*’*s welfare in relation to* ___________ (*issue under discussion*)? *Do you think farmers see this as a concern*?

► (*Getting beyond cost* &*money*)

*Cost is naturally a limiting factor for those in agribusiness but if money were the only driver*, *there would be a lot less farmers*! *What else motivates farmers to adopt more animal welfare*-*friendly practices*?

III. **Discussion of approaches to improve welfare** &**the Scheme using** ‘**start**, **stop**, **continue**’ **evaluation**: (**15 min**)

· The focus group facilitator will explain:

“*The laminated table just distributed relates to the current Scheme for Suckler Herds*’ (*the* ‘*Suckler Scheme*’) *and the 7 measures it involves* (Table 3).

a) *If a new financial welfare scheme were to be repeated in the future*, …

*Which measures from the current scheme should be used* (*continue*)?

b) *Which measures from the current scheme should be discontinued* (*stop*)?

c) *What other measures should be added to improve farm animal welfare* (*start*)?

*This scheme which involves payments awarded for meeting the 7 criteria represents one mechanism to improve the welfare of beef cattle*.

d) *In your opinion whatothermechanisms could be taken to improve farm animal welfare*?

e) *It has been proposed that education*/ *training is one of the most effective ways to improve animal welfare*. *What are your thoughts on this*? *What are your views of the existing information and training resources available* (*enough or not*? / *appropriate to farmers*’ *needs or not*?)?

IV. **Closing** (**5 min**)

· Participants invited to mention anything else of relevance they feel has not been covered yet

· Closing remarks (AD’s contact details/ participation in further studies/ where to find results of research study)

· Thank the participants and give vouchers and ensure signed receipts.

## Competing interests

The authors declare that they have no competing interests.

## Authors’ contributions

All authors contributed to the study design and participated in writing the paper. All authors have read and approved the final manuscript.
